# Secondary dispersal driven by overland flow in drylands: Review and mechanistic model development

**DOI:** 10.1186/2051-3933-2-7

**Published:** 2014-04-17

**Authors:** Sally E Thompson, Shmuel Assouline, Li Chen, Ana Trahktenbrot, Tal Svoray, Gabriel G Katul

**Affiliations:** Department of Civil and Environmental Engineering, UC Berkeley, 661 Davis Hall, Berkeley, California 94720 USA; Soil, Water and Environmental Sciences, A R O – Volcani Center, Bet Dagan, 50250 Israel; Division of Hydrologic Sciences, Desert Research Center, Las Vegas, Nevada 89119 USA; Nicholas School of the Environment, Duke University, Box 90328, Durham, North Carolina 27708 USA; Geography and Environmental Development, Ben-Gurion University of the Negev, Be’er Sheva, Israel; Pratt School of Engineering, Duke University, Durham, North Carolina 27708 USA

**Keywords:** Seed dispersal, Overland flow, Semi-arid, Eulerian, Lagrangian, Modeling

## Abstract

Seed dispersal alters gene flow, reproduction, migration and ultimately spatial organization of dryland ecosystems. Because many seeds in drylands lack adaptations for long-distance dispersal, seed transport by secondary processes such as tumbling in the wind or mobilization in overland flow plays a dominant role in determining where seeds ultimately germinate. Here, recent developments in modeling runoff generation in spatially complex dryland ecosystems are reviewed with the aim of proposing improvements to mechanistic modeling of seed dispersal processes. The objective is to develop a physically-based yet operational framework for determining seed dispersal due to surface runoff, a process that has gained recent experimental attention. A Buoyant OBject Coupled Eulerian – Lagrangian Closure model (BOB-CELC) is proposed to represent seed movement in shallow surface flows. The BOB-CELC is then employed to investigate the sensitivity of seed transport to landscape and storm properties and to the spatial configuration of vegetation patches interspersed within bare earth. The potential to simplify seed transport outcomes by considering the limiting behavior of multiple runoff events is briefly considered, as is the potential for developing highly mechanistic, spatially explicit models that link seed transport, vegetation structure and water movement across multiple generations of dryland plants.

## Introduction

Seed dispersal, or the process by which seeds are *mobilized*, *transported* and eventually come to rest prior to germination [1] forms a critical stage in reproductive biology. It is the main process that determines population migration rates, invasion dynamics, patterns of gene flow and spatial organization of the landscape [2]. Seed dispersal is diverse, encompassing both *biotic* (animal mediated) and *abiotic* (physically mediated) processes [3]. Several abiotic dispersal processes such as wind and water-driven seed dispersal are amenable to a theoretical description, using well-established principles from fluid mechanics to describe the seed dispersal as *inertial particle transport in turbulent flows*[4, 5]. For example, the specific problem of seed dispersal by wind over homogeneous, closed vegetation canopies has been sufficiently advanced to pemit estimates of transport distances over which seed populations are dispersed [3, 6–10]. These solutions depend upon the properties of the dispersed seeds, wind statistics above the vegetation canopy, the seed release height, and the vertical distribution of the canopy leaf area.

This paper modifies the theoretical treatment of seed dispersal to account for the secondary dispersal of seed by overland flow in spatially patchy drylands [11]. The seeds of dryland plants usually lack adaptations that promote long-distance primary dispersal [12]. Seeds undergoing primary dispersal (from plant to the ground) travel only short distances. The distances travelled by fallen seeds (secondary dispersal) have a high probability of being much longer than those travelled in primary dispersal. Thus, secondary dispersal determines the locations in which seeds come to rest and germinate [13–15]. Water [12, 15–19] and wind [13, 20–22] are both abiotic seed transporting vectors for secondary dispersal in drylands. Their relative importance remains a subject of active research, and is likely controlled by the overlap between dispersal periods and the rainy season. While at least one theoretical treatment of secondary dispersal by wind in drylands has been proposed [23], no attempts to develop a mechanistic model for seed dispersal in overland flow have yet been made. Yet, recent increases in studies exploring water-driven dispersal in drylands [15–19], in modeling overland flow processes in patchy landscapes [24, 25], and in the broader realm of water dispersed seed dynamics (hydrochory) [26] suggest that the time is ripe to develop such theory.

Dispersal of seed via overland flow is clearly a form of hydrochory, and could incorporate both nautochory (the dispersal of floating seeds at the surface of a water column) [27] or bythisochory (dispersal of non-floating seeds along the base of a water column) [28]. Dispersal in overland flow, however, has characteristics that differentiate it significantly from typical hydrochory along a stream network or within wetlands. These characteristics include the following mechanisms: (i) the initiation of dispersal relies on the occurrence of relatively infrequent intense rainfall events that generate sufficient overland flow to move seeds (by comparison, in most streams and rivers, flow is perennial or nearly so, and the initiation of hydrochory relies on primary or secondary transport of seeds to the flow channel); (ii) the termination of dispersal is dictated by seed trapping or the cessation of overland flow (by comparison, stranding of seed on river banks or floating vegetation, or burial of seeds that change their density over time are the primary modes of termination of in-channel hydrochory) [29, 30]; (iii) flow is not confined to the vicinity of the channel network, and consequently (iv) overland flow can lead to long-distance seed dispersal, over shorter length-scales but also a less-constrained areal extent than hydrochory within rivers.

This study proceeds in three parts: (i) a review of the relevant flow generation and seed characteristics that influence secondary dispersal by overland flow; (ii) extension of existing seed transport theories to overland flow in sparse canopies, and an illustration of theoretical results from this extension; and (iii) a discussion of the implications of these results for spatial ecology in drylands.

## Review

### Overland flow generation in drylands

Bare soils in drylands are directly exposed to rain impact and sunlight, leading to the formation of structural and sedimental soil seals [31], and biological soil crusts [32]. Together, seals and crusts form a compacted, disturbed layer at the soil surface, characterized by low saturated hydraulic conductivity [33, 34]. They drastically reduce soil infiltrability and lead to the formation of infiltration-excess overland flow [35–39]. Conversely, vegetated patches are characterized by high surface roughness [25, 40] and high infiltration rates [41], and inhibit the formation of overland flow [42]. The patchy structure of drylands therefore leads to spatially fragmented patterns of overland flow initiation [43]. Pervasive microtopographical variation creates further spatial distinctions between narrow, deep and fast-flowing zones where flow velocities can be 2-7 times higher than their areal averaged counterparts; to broad, shallow, slow-moving zones where flow velocities approach zero [24, 44–46]. The immediate generation of runoff from rainfall events, specifically those of sufficient intensity to exceed local infiltration capacities [42, 47] leads to surface runoff that is highly intermittent through time. Theoretical treatments of the seed-transporting flow field cannot ignore the spatial patchiness in flow initiation and flow characteristics.

The Saint Venant equations (SVE), named after the mathematician and hydraulic engineer Adhémar Jean Claude Barré de Saint-Venant, can be expanded to include spatially variable roughness and lateral source/sink terms. These equations are based on the depth-averaged Navier-Stokes equations and describe surface runoff under the (almost always reasonable) assumption of shallow flow [25, 40]. In the simplest one-dimensional case along the longitudinal direction (that is, the direction of mean flow), the combined continuity (or conservation of water mass) and Saint Venant equations are given as:1

where *t* is time, *x* is a downslope or longitudinal distance; *h* is the water depth, *q*_*x*_ (= *V h*) is the flow rate per unit width along direction *x*; *V* is the depth-averaged velocity; *g* is gravitational acceleration; *P(x,t)* is the rainfall, allowed to vary through space to account for e.g. local changes such as throughfall within vegetated sites or larger-scale changes associated with spatial variation in the rainfall field; *I(x,t)* is the infiltration rate that varies throughout the storm and as a function of vegetation cover; *S*_*o*_ is the ground slope (and may be zero) and *S*_*f*_*(x)* the friction slope, which reflects the total energy head losses per unit flow length due to simultaneous ground friction and drag imposed by the vegetation. The time-dependence of infiltration can be accounted for via standard or seal-layer specific formulations [42, 48]. It is assumed that time scales responsible for variations in *h* and *V* are much faster than the timescales over which biomass changes. This timescale separation between flow and biomass changes allows the spatial distribution of vegetation to be specified as a function of location *x*. It is also assumed that parameterizations are available to relate the friction slope, rainfall and infiltration properties to the vegetation characteristics [25]. These equations can be solved for the space-time variations of *h, q*_*x*_ (and hence *V)* provided a ‘closure’ for *S*_*f*_ is formulated. In general, such a closure relates *S*_*f*_ to *V* and *h* via a friction factor *f*, for example:2

The friction factor *f* varies with surface roughness, *h*, and with the bulk Reynolds number, **Re**_**b**_ = *Vh/ν*, where *ν* is the kinematic viscosity of water (about 10 times smaller than its air counterpart). When the flow is fully turbulent, **Re**_**b**_ >500 and Manning’s equation is used to link *f* to the Manning roughness coefficient, which varies only with the surface properties [49]. However, for laminar flow conditions, **Re**_**b**_ < 500 and *f* varies with **Re**_**b**_. The determination of *f* for vegetated patches is complicated by other factors due to the presence of localized drag forces at the vegetation-water interface (potentially larger than the ground shear stress), but which lie outside the immediate scope of this study. However, analytical formulation linking *f* to vegetation attributes such as leaf area index, leaf drag, and water level have been derived for the large **Re**_**b**_ case [50]. Flow disturbances induced by rainfall events can impact *f* and even the generation of turbulent kinetic energy that increase the velocity variance around *V*, although these effects are rarely considered in hydrologic models. The solution to these equations also requires that the flow is sub-critical (i.e. Froude Number **Fr** < 1) thereby avoiding formation of hydraulic jumps along with their associated energy losses not considered in *S*_*f*_.

**Figure 1:**
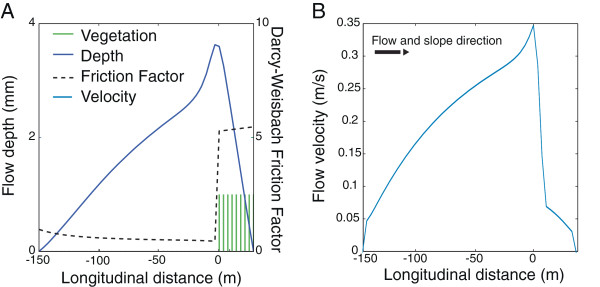
**Characteristics of overland flow in a patchily-vegetated semi-arid hillslope, as simulated via the Saint-Venant equations. (A)** Illustration of the flow depth profile (dark blue line, on the left hand axis) and the friction factor (dashed black line, on the right hand axis) for a 150 m bare zone adjacent to 30 m of vegetation (green lines), on a 5% slope, at the end of a 30 minute, 5 cm/hr storm. **(B)** Profile of the flow velocity for the same conditions. Longitudinal distance in both plots normalized so that 0 m occurs on the upslope edge of the vegetation.

Given varying boundary conditions associated with the storm temporal evolution and the land surface properties, Equation (1) may be solved, and the space-time varying fields of flow depth, flow (or velocity) and friction factor computed both in the non-vegetated and vegetated sites. A snapshot of these fields in one dimension are shown in Figure 1, illustrating the large increase in the friction factor associated with the presence of vegetation, and the peaks in flow depth and velocity that occur at the bare soil – vegetation boundary in response to this change. These results are used to drive Lagrangian seed dispersal models, as described in the next section.

### Seed dispersal in overland flow

One body of research addressing seed dispersal in overland flow has viewed secondary dispersal as a *negative* outcome: for instance inhibiting revegetation efforts in degraded landscapes [51, 52], preventing plant colonization of hillslopes [19] and resulting in recruitment in environments that represent sub-optimal seedling habitat [13]. These studies report the rate of seed loss due to overland flow, rather dispersal locations. Another body of research recognizes that secondary dispersal by water may be significant for determining the structure and functioning of dryland ecosystems [13, 53–60]. These studies identify species zonation, seed trapping and transport efficiencies, and explore the long-term and large-scale outcomes of dispersal by water.

Studies of seed movement in overland flow illustrate several pertinent features of seeds that influence their movement in surface water. For example, Table 1 reports the physical properties of seeds from 14 desert plants, as well as the average seed properties of an assemblage of 83 desert species from Spain. Only one of these seed types is denser than water. The seeds of 60 species in the Loess Plateau region of China were also all found to be buoyant [19]. Thus, provided there is sufficient water depth available (i.e. *h > d*, where *d* is a seed size), seed will float and move with overland flow.

**Table 1:** **Physical properties of seeds from dryland species, and their estimated terminal velocities in water**

Seed type	Mass (mg)	Dimensions (mm by mm)	Density (kg m^-3^)	Terminal velocity in water (ms^-1^)
*Prosopsis flexuosa*	24^1^	6.0–6.6 × 1.9–2.2^2^	906	8 (upwards)
*Larrea sp.*	2.3^1^	6.0 mm spheroids^3^	10.6	77 (upwards)
*Atriplex lampa*	0.62^1^	6-12 × 5 – 12^4^	1.38	140 (upwards)
*Trichloris crinita*	0.18^1^	4 × 0.5^5^	180	28 (upwards)
*Sporobolus cryptandrus*	0.07^6^	0.68 × 0.40^6^	643	0.4 (upwards)
*Chenopodium papulosum*	0.25^6^	0.94 × 0.82^6^	396	1.2 (upwards)
*Pappophorum spp. a*	0.35^6^	1.32 × 0.47^6^	1200	0.76 (downwards)
*Digitaria californica*	0.40^6^	1.67 × 0.98^6^	250	4.5 (upwards)
*Parthenium hysterophorus*	0.42^6^	2.45 × 1.18^6^	123	11 (upwards)
*Phacelia artemisioides*	0.50^6^	1.46 × 1.02^6^	330	3.1 (upwards)
*Setaria leucopila*	0.75^6^	1.18 × 1.04^6^	588	1.2 (upwards)
*Plantago patagonica*	0.80^6^	2.27 × 0.98^6^	367	7.1 (upwards)
*Pinus pinea*	443^7^	Vol: 1067 mm^3 7^	415	
*Erica multiflora*	0.07^7^	Vol: 0.16 mm^3 7^	438	
Average of 83 Spanish desert seeds	35.28^7^	Vol: 67.91 mm^3 7^	520	

Data sources in table: ^1^[55], ^2^[61], ^3^[62], ^4^[63], ^5^[64], ^6^[65], ^7^[52].

Seed dispersal by overland flow is influenced by other seed characteristics. Larger seeds are less likely to be mobilized [15, 18, 19, 51, 52]. More intense storms are more likely to mobilize seeds [19]. Several species have adaptations such as awns, hairs, and pappi that enhance seeds trapping [51], some are preferentially dispersed into cracks [15], and some excrete mucilage when wet [15, 19, 51, 54]: adaptations that tend to prevent dispersal by water (although some studies suggest that mucilage increases seed buoyancy and promotes dispersal in runoff [54]).

In summary, several empirical studies suggest that: (i) seeds will float; (ii) transport initiation is a critical stage of dispersal; (iii) transport initiation is less likely for larger seeds; and (iv) adaptations that increase the likelihood of seed trapping influence transport. These common findings provide the minimum input to the development of theoretical descriptions of seed transport in overland flow.

## Seed dispersal in overland flow

### Inertial particle transport by moving fluids

Seeds in a fluid flow are accelerated by both gravitational and drag forces. Gravitational acceleration is reduced by buoyancy to give a reduced gravitational field *g’*:3

where *ρ*_*f*_ is the density of the fluid (water here), and *ρ*_*p*_ is the density of the seed (particle) when wet. Note that where the density of the seed is less than the density of water, as appears to be the case for the vast majority of seeds considered here, this implies a negative reduced gravity, i.e. a positive buoyancy and floating seeds.

The strength of drag forces acting on the seed is proportional to the difference in the seed and fluid velocities, according to the equation:4

where *C*_*d*_ is the drag coefficient, *A* the surface area of the seed, *m* is the seed mass, **V**_**f**_ the velocity vector for the moving water and **V**_**p**_ the velocity vector for the seed particle.

Seeds with adaptations that increase their surface area such as wings, therefore experience high drag forces for relatively small velocity differences, and these cause the velocity of the seed to approximate that of the fluid (i.e. the seed is well-coupled to the moving water). Smaller surface areas do not couple seed and fluid velocities tightly, and changes in seed velocity will lag behind changes in fluid velocities for these particles [66].

The seed acceleration can be estimated from the drag forces at any spatial location, and integrated along the seed’s path to yield its velocity and displacement. This approach relies on coupling the Eulerian flow statistics (which determine **V**_**f**_ at any point in space or time) to the Lagrangian description [67] of seed motion. It is therefore known as the Coupled Eulerian Lagrangian Closure model, or CELC [5, 10, 68]. Boundary conditions defining seed mobilization and the termination of transport must be imposed for specific dispersal problems [66, 69]. Note that this framework does not account for cases where the seed or its **V**_**p**_ interact with or alter the fluid velocities.

CELC may be run for an ensemble of seeds through Monte Carlo simulations of the travel paths, yielding a probabilistic description of seed transport distances from a single seed source position. The resulting probability density function (PDF) of seed originating from any individual point is known as the *dispersal kernel*. Kernels provide a parsimonious description of dispersal and can be directly incorporated into spatial models of the plant population, as has been done in a number of recent studies [70, 71].

In the overland flow problem, the dispersal kernel is spatially heterogeneous and will vary for each potential point of seed release depending on the flow experienced locally at that point, and the downslope distance to vegetated patches that intercept flow and seeds. This situation offers three possible approaches for the spatial representation of dispersal: (i) a description of only mean transport lengths initiated from every point in space (at a manageable computational cost, but at the cost of preserving only one moment of the dispersal kernels); (ii) the generation of an individual dispersal kernel for every potential release point, which can then be spatially summed to obtain the final distribution of dispersed seeds throughout the domain (comprehensive, but with a high computational cost); or (iii) in landscapes with strong spatial organization (i.e. a consistent length-scale between vegetated patches) and strong trapping of seed by vegetation, multiple dispersal events may cause the cumulative dispersal lengthscales to converge. In these landscapes, it might be possible to generate an effective dispersal kernel that could be used to approximate seed transport as a low-dimensional basis for modeling - especially if such models are subjected to spatially periodic boundary conditions.

Here the first possibility is explored using CELC to estimate mean seed transport distances given the location of the seeds following primary dispersal. As explored in Section Adapting CELC for Buoyant Seed Transport, the ensemble mean of all seed trajectories provides a reasonable description of the population-level transport because the low velocities of overland flow minimize the potential for turbulent spreading of dispersed seeds (in comparison to wind-dispersal in forested landscapes). A similar approach was adopted by Trakhtenbrot et al. [72] to address the characteristics of seed dispersal from uniform canopies in heterogeneous (hilly) terrain. The cumulative effects of multiple storms on seed distribution are also explored to assess the feasibility of the third case. Although it is not implemented in this study, additional drivers of variability in dispersal length-scale could be readily coupled to CELC and used to drive the definition of spatially-varying kernels for heterogeneous landscapes. Naturally, these additional drivers are site- or problem-specific and thus lie outside the scope of this review.

### Adapting CELC for buoyant seed transport

Three adaptations are introduced to modify the CELC framework from its original formulation for wind dispersal over homogeneous canopies to seed dispersal in overland flow. The first is to assume no net vertical seed transport. That is, once the flow depth is large enough, buoyant seeds float on the surface, and fluctuations in the seeds' vertical position simply follow the flow depth. The second adaptation is to account for time variation in the Eulerian flow velocities such that they can be inferred from *V* and *h*. In the case of wind dispersal, individual seed flights are short compared to the 30-60 minute periods on which wind statistics are usually pseudo-steady [70]. No such timescale separation exists in the case of overland flow. The third adaptation is to account for spatial dependence of the Eulerian statistics, driven by the spatially patchy nature of runoff in drylands. The modified CELC framework is referred to as the “Buoyant – OBject CELC” model (BOB-CELC). It simplifies CELC by neglecting vertical velocity fluctuations, at the expense of resolving the full space-time variation of the other velocity components. Hence, the strength of BOB-CELC is that unlike the horizontal homogeneity of vegetation and flow assumed in current CELC treatments of dispersal by wind, for overland water flow the vegetation and flow heterogeneity effects are explicitly incorporated.

To reflect the observation that transport initiation was most probable for small seeds, for transport to be initiated, *h* at any *x* must exceed the seed diameter *d*. In practice, this requirement imposes a rainfall intensity threshold for seed mobilization that is specific to the seed properties, storm characteristics, slope, surface roughness and infiltration capacity of a site. In the simulations here, high intensity storms (2.5 – 7.5 cm/hr) of relatively short duration (5 – 15 minutes) are considered: in part because these storms provide a clear illustration of the use and results from BOB-CELC, but also because such storms occur (a) on sub-annual timescales in the semi-arid substropics [73], and because (b) a global biogeographic analysis of large-scale organization in dryland vegetation suggests that it is strongly associated with tropically dry regions with a pronounced and intense wet season [74]. In practice, of course, not all storms will be capable of moving all seeds. A general constraint on the mobilization of seeds is that:5

where *P* is the rainfall intensity, *f* the local infiltration capacity, *D* the storm duration and *d* the seed diameter. This condition is necessary in all situations, and sufficient in the limit of topographically flat sites where the ponded depth *h* is not diminished by lateral flows, nor enhanced by flow concentration.

Similarly, transport is terminated either when the flow depth declines below the seed diameter, or the seed is trapped. The seed displacement equations (referenced to the seed release position) adopt the form:6

where *u*_*p*_ and *v*_*p*_ are the particle velocities in the *x* (longitudinal) and *y* (lateral) directions, evolving due to the action of drag (Equation 4). The local *turbulent* flow velocities in the *x* and *y* directions, *u* and *v*, are computed from the Eulerian flow field in conjunction with assumed scaling of the flow statistics by summing the previous turbulent flow velocity at any location with its evolution along the fluid path, given by:7

Here *u*_*B*_ and *v*_*B*_ are the vertically-averaged bulk velocities in the _*x*_ and _*y*_ directions which constrain the turbulence statistics and are obtained from the Eulerian flow field (Equation 1), the *α* and *β* terms are estimated using the solution of Thomson [67], and the terms *dξ*_*x*_ and *dξ*_*y*_ are normally distributed stochastic increments with mean zero and standard deviation of *dt*, that reflect the turbulent velocity fluctuations in the *x* and *y* directions. Under the assumption that seeds rapidly reach the surface of the flow and that the vertical turbulent fluctuations are strongly damped by presence of a shallow free surface flow, the Thomson solution simplifies to:8

Here *ε* is the mean turbulent kinetic energy dissipation rate (computed from the flow Reynolds number using k-epsilon scaling), and *C*_*O*_ is the Kolmogorov constant for the Lagrangian structure function, taking a value of 3.125. The magnitude of the fluctuations scales with the standard deviation of the longitudinal *u*_*B*_ and lateral *v*_*B*_ velocities, *σ*_*u*_ and *σ*_*v*_[75]. Empirical observations in open-channel flow suggest that these standard deviations, along with the standard deviation of vertical fluctuations, *σ*_*w*_, included for completeness, scale with the local shear or friction velocity u* as follows [76]:9

**Figure 2:**
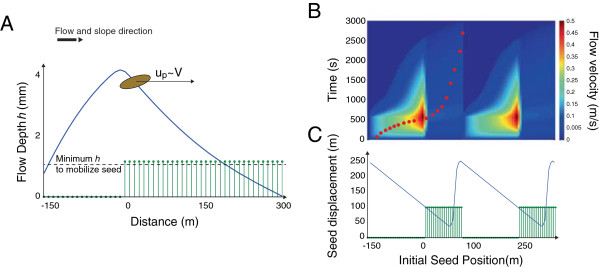
**Schematic of seed transport behavior within overland flow as modeled by BOB-CELC. (A)** Conceptual diagram of assumed seed transport mechanism of simple advection at the surface of the overland flow, for all regions in which the flow depth is sufficient to mobilize the seed. **(B)** Illustration of space­time plots of the flow velocity, with a 1­D Lagrangian seed path illustrated by red dots. **(C)** Rather than a dispersal kernel, a spatially distributed set of seed displacements as a function of the starting position is generated through advective transport.

These relations were derived for planar homogeneous boundary layer flows where the turbulence is fully developed so that mechanical production of turbulent kinetic energy scales with u*^3^. Equations 4 - 9 in conjunction with the Eulerian velocity fields (obtained from solution of Equation 1) form the BOB-CELC model. BOB-CELC is solved by integrating the seed transport equations throughout the space-time field of the velocity as illustrated for a one-dimensional case in Figure 2. The Lagrangian equations that form BOB-CELC are greatly simplified compared to the three-dimensional atmospheric flow scenario for which CELC was originally derived. In the three dimensional case, vertical velocity fluctuations exert a dramatic influence on the particle motion. Here it is assumed that vertical turbulent fluctuations do not exert a significant influence on the motion of a fluid particle confined to the surface of the flow (and hence to any seed motion). The scaling in equation (9) provides a first order rationale for this simplification: lateral and longitudinal fluctuations are leading order terms compared to the vertical fluctuations, which are confined to a small range in *h*, where the vertical velocity variance distribution within *h* is significantly damped by the presence of the no slip boundary at the ground and free water surface at the top.

To test whether the assumption that seed trajectories are well represented by the mean seed trajectory (and displacement distance) is valid, we ran simulations where 50 seeds were released at four locations: upslope of a vegetated patch, at the patch boundary, within the vegetated patch and downslope from the vegetated patch. The seeds were routed through BOB-CELC for a 5 cm/hr, 5 minute long storm, and the resulting variance in the spread of seed travel distances computed for seed from each initial location. The variance in these dispersal kernels was, on average 2 mm, with the greatest variance being only 4 mm – in comparison to the average transport distance from each location, which was on the order of 75 m. The five orders of magnitude difference between the transport length and the spread in the seeds suggests that the seed motion is overwhelmingly kinematic and quasi- deterministic in these low-turbulence systems.

This result requires discussion, since the finding of minimal variance in seed dispersal length-scales appears counter-intuitive. This result, however, should not be interpreted as indicating that seed transport in overland flow is entirely deterministic. Instead, it indicates that turbulence within the flow trajectories is not the major source of variance in dispersal length-scales in shallow overland flow. This contrasts markedly with wind dispersal, in which turbulence is a major driver of variability in dispersal length-scales. However, the distinction between the two cases can be readily interpreted in terms of the differences in the Reynolds numbers of the flow: on the order of 10^0^-10^2^ for shallow overland flow, and on the order of 10^5^-10^6^ for wind dispersal: this suggests that travel variances due to turbulence *should* be many times smaller in overland flow than in wind dispersed cases. However, other sources of variability in dispersal length-scales can and should be considered when modeling seed dispersal in overland flow. Two likely sources of such variability include the time at which dispersal is initiated (the results here assumed simultaneous mobilization of all seeds at a given location), and variability in the termination of transport by the trapping of seeds. Each of these sources of variability can be readily incorporated into BOB-CELC. However, the physical basis for the parameterization of stochastic transport initiation and termination of seeds remains unclear, and further research is required. For this reason, we have retained only the most elementary descriptions of a single transport initiation time, along with a highly simplified treatment of seed trapping as described below.

Physical trapping of seeds by soil and vegetation is an additional mechanism for terminating secondary transport, although there is little empirical data as to the magnitude and nature of the trapping. For simplicity, it is assumed that there is a uniform probability of seed trapping per unit distance a seed is transported in a vegetated patch, e.g. probability *Π* of trapping per 1 m distance travelled. The rationale for this simple, and purely phenomenological assumption is that the further the seed is transported within the patch, the greater the probability of being intercepted by a roughness element and being trapped: other drivers of trapping probability such as seed velocity or flow depth are not addressed in this simple model. This imposes a sink given by:10

applied at every timestep while the seed is located within a vegetated zone. Similar probabilistic approaches could be used to describe the effect of seed adaptations that promote trapping. Without detailed data about trapping due to vegetation morphology or seed characteristics, these effects cannot be explored in detail.

### Effects of storm, vegetation, hillslope and seed characteristics

To explore the effects of storm, vegetation, hillslope and seed characteristics on transport in a synthetic patchy landscape, a suite of flow scenarios on a linear hillslope covered with two repeating units consisting of a region of bare ground and a large vegetation patch is developed. To solve Equation 1, the roughness and infiltration parameterization are taken from previous studies [25]. These scenarios represent seed transport associated with e.g. banded vegetation in drylands [77]. The size of the vegetated patch is varied as well as the contrast in the infiltration rates between bare and vegetated patches, the slope angle, and storm properties. The boundary conditions applied to the flow were a no-flux boundary condition on the upslope edge of the first bare area (i.e. *u*_*B*_ *= 0*) and a constant-flux boundary condition on the downslope edge of the second vegetation patch (i.e. *du*_*B*_*/dt = 0*). The no-flow boundary condition can either be considered to represent the condition at a hillslope divide, or, more generally, the condition on bare soils downslope of a vegetated patch that prevents significant lateral discharge of runoff. The constant-flux boundary condition is applied to allow runoff water to evacuate the domain. Model results are presented showing only the second of the repeating units (bare-vegetated), allowing for edge effects from the upslope boundary condition to be dampened.

**Figure 3:**
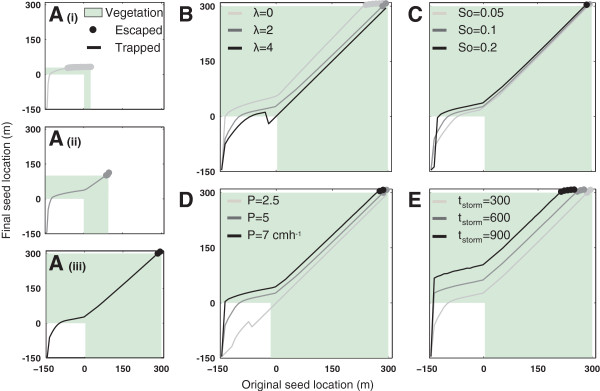
**Sensitivity of BOB-CELC displacements to storm and landscape properties showing the mean final seed location as a function of its initial position. (A)** Increasing vegetation patch sizes 30 m (i), 100 m (ii) and 300 m (iii). The dots on all panels indicate that seeds initiated from this position ‘escape’ from the vegetated patch in the simulation, all other seeds are trapped within the patch at the end of the storm. **(B)** Increasing the infiltration contrast between bare and vegetated sites (λ = 0, 2 and 4) **(C)** Increasing land surface slope (S_o_ = 5, 10 and 20%). **(D)** Increasing rainfall intensity (P 2.5, 5 and 7 cm/hr^-1^ shown, P = 1.1 and 1.5 cm/hr^-1^ also tested). **(E)** Increasing the duration of the storm (tstorm = 300, 600 and 900 seconds). Baseline conditions are for a 150 m long bare patch, P = 5 cm/hr^-1^, t_storm_ = 300 seconds, λ = 2, S_o_ = 0.1 and a 300 m vegetated patch.

Figure 3 illustrates the results of these model runs. Smaller vegetated patches increase the mean flow depth and velocity within the patch, increasing transport length scales and suggesting that patch scale is likely to influence the likelihood of seed transport through, or from, the patch to its downslope edge. If no infiltration occurs within vegetated patches, the transport length scales increase through the patch due to a ‘backwater’ effect caused by the low flow velocities in the vegetation patch, that prolongs the duration of flow. Intermediate infiltration rates prevent flow accumulation in the vegetated patch, leading to near constant transport length scales. High infiltration rates disconnect the overland flow within vegetated patches and prevent seed transport, in agreement with experiment, see [78]. Increased slopes, storm intensities and storm durations promote long transport length scales and increase the probability that seeds escape vegetated patches [19, 51, 52].

**Figure 4:**
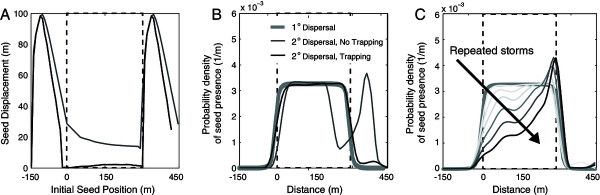
**Sensitivity of seed transport behavior to different storm properties. (A)** seeds displacement distance for a case with no trapping (gray) and a case where seeds are trapped by vegetation at a rate of 50% per 1 m travelled representing the two ‘end­ member’ cases. Trapping leads to minimal transport within the vegetated band. **(B)** Probability distribution of the locations of seeds dispersed from the vegetated patch. Note that this is *not* a dispersal kernel (which shows the probability of movement distances conditional on an initial location), rather this is a probability distribution of the proportion of the seed bank generated by the vegetated patch being present at any given point. This distribution was derived using a primary dispersal kernel based on WALD assuming an average dispersal distance of 10 m. Secondary dispersal was examined with no trapping of the seed in the vegetation (gray lines) and with 50% seeds trapped per 1 m travelled (black lines). **(C)** Effect of repeated storms on seed distribution in the vegetated patch. The outcome of 10 storms is shown here, for the trapping case. It leads to a distinct bias in seed accumulation at the downslope edge of patches. In all simulations, P = 5 cm/hr^-1^, t_storm_ = 300 seconds, λ = 2, S_o_ = 0.1 and the vegetated patch is 300 m long.

The displacement plots in Figure 3 provide a perspective on transport probabilities, but (i) omit the potential effects of seed trapping; and (ii) do not indicate the likely spatial distribution of seeds following secondary-dispersal. To account for the former, we reproduced the 10% slope, 300 m vegetated patch results with an assumed trapping probability of 50% per meter of transport within vegetated sites. To develop a final seed density distribution, we used the close analytical approximation to the CELC model, the WALD (or inverse Gaussian) distribution [6, 70] to generate a distribution of seed initial positions following primary dispersal by wind (with an assumed mean dispersal length scale of 10 m) for both the vegetated patches. We then applied BOB-CELC to account for the effects of secondary dispersal following this primary WALD dispersal event, with and without seed trapping. Figure 4 shows the results, indicating that trapping would greatly inhibit seed transport through and within vegetated bands (Panel A). Secondary dispersal in an intense, half-hour storm with no seed trapping generated a downslope dispersal of seeds to bare sites, but led to almost no seed transport if trapping occurred at the 50% per meter rate (Panel B). Repeated storms (assuming the same storm conditions) eventually exported all seed from the site in the absence of trapping, but caused a bias in seed distribution towards the downslope edge of vegetated patches if vegetation was effective at trapping seed (Panel C). Although few experimental studies have explored this process, Emmerson et al. [79] monitored secondary dispersal over a 9 month period, finding that trapping in areas with low slopes or significant seed burial, often associated with vegetated sites, effectively ended secondary dispersal in this species. Qualitatively, this suggests that the behavior modeled in Figure 4 is reasonable, although quantitative modeling of trapping requires further investigation.

### Spatial consequences of seed dispersal in runoff in drylands

**Figure 5:**
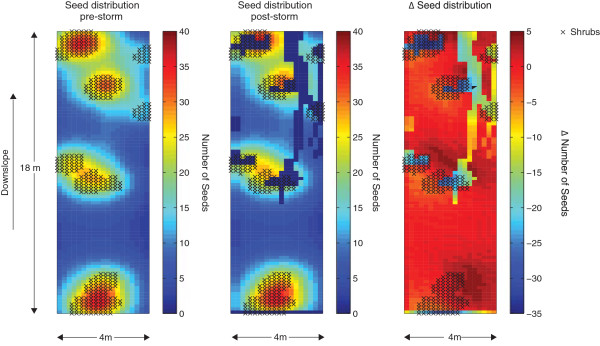
**Application of BOB**-**CELC to hypothetical seed distributions during a real storm event.** The left hand panel illustrates initial seed distributions on a steep, patchy landscape, assuming seed were dispersed from shrubs with a mean dispersal distance of 1 m. The central panel shows the seed distribution after 1.5 hours of a storm that produced surface runoff as simulated, calibrated and validated by [24]. The right hand panel indicates the change in seed numbers induced during the storm.

The simple cases considered above suggest that repeated dispersal of seeds by runoff in drylands will lead to one of several outcomes: (i) export of seeds from the domain and thus seed “loss”; (ii) preferential accumulation of seeds at the downslope edge of vegetated patches; or (iii) transport of seed from one patch to another patch. The first two cases find literature support. For example, formation of micro-topographic depressions outside shrub canopies routed water away from shrubs, and prevented seed trapping in olive scrub in Ethiopia [78], a clear example of seed “loss”, which is likely to lead to seed deposition in bare sites where predation rates are elevated, probability of germination is suppressed and nursery- plant effects of the mature canopy are unavailable [80–82]. Conversely, observations in banded vegetation (which traps overland flow effectively) in Mexico suggested preferential growth of vegetation in the downslope edge of the band [56, 83, 84]. Simulations of landscape dynamics suggest that downslope seed transport may be a stabilizing mechanism that is required to explain the slow or absent rates of upslope migration in vegetation bands [71, 85]. Conversely, average dispersal distances in overland flow were too short or too strongly directed along degraded areas or animal tracks to allow significant patch- to-patch seed dispersal for an arid-land daisy species [86]. Nonetheless, patch-to- patch seed transmission should be in principle feasible. Figure 5 illustrates the coupling of BOB-CELC with a 2D, validated model of runoff production on a slope, presented in Chen et al. [24]. This runoff model accounts for observed surface roughness, soil seal-layer formation, shrub locations and microtopographic variations, and was driven by observed rainfall. Observed locations of shrubs were used to initialize seed locations with the WALD model, assuming a mean dispersal distance of 1 m. BOB-CELC was run assuming small seeds (1 mm diameter) and neglecting trapping. Two patterns emerged: significant loss of seeds in regions of concentrated flow (shown by the obvious regions of channel formation), and a broad pattern of seed displacement from areas near shrubs to zones approximately 5 m downslope. Both are broadly consistent with the seed transport patterns observed during overland flow events in arid systems in Australia [79, 86]. The modeling suggests that patch-to-patch transmission may be feasible under situations where: (i) storms are long enough and intense enough to induce transport distances comparable to the inter-patch spacing (<10 m for the storm shown in Figure 5), or where multiple storms occur, allowing repeated transport; and where (ii) connectivity between patches is feasible topographically and hydrologically. The major obstacle to between patch connections as shown in Figure 5 is likely to be the formation of concentrated flow paths that can effectively bypass vegetated patches, or transport seeds through the patch effectively. Other landscape features – for example the presence of ant mounds and activity – which are not explicitly accounted for in these model runs, could also potentially influence dispersal (but could readily be incorporated into the model as mapped regions of disturbed soil with specific trapping capacities) [87–89]. Seed tagging, or DNA analyses could be used to ascertain the important of seed transport in runoff for maintaining genetic connectivity between patches. Critically, the simulations also indicated that for this soil type, almost no overland flow would have been generated under the modeled rainfall conditions in the absence of soil seal layer formation. There is thus likely to be a strong relation between seal formation and secondary dispersal by overland flow.

The results show that unsurprisingly, secondary dispersal by overland flow is highly anisotropic and only transports seeds downslope. The detailed modeling approach presented in Figure 5 can be used to examine whether secondary dispersal in overland flow could result in ‘directed’ dispersal, that is preferential dispersal to habitats that may favor seed establishment and recruitment to adulthood [90, 91]. Assuming that in drylands vegetation patches are indicative of favorable habitat, the preliminary results presented here suggest that the answer will depend on storm parameters such as intensity and duration – that might determine whether the flow will be channeled around the vegetation - as well as on the patch spatial organization.

While the results so far provide a mechanism to describe seed dispersal and thus to link generations of plants in space, we have not explicitly simulated the evolution of spatial vegetation patchiness in drylands. Heuristically, downslope trapping of seeds suggests that the anisotropic dispersal could be prescribed with an effective dispersal kernel that localizes the modal dispersal at the bottom edge of vegetated patches. An analogous approach has been used previously to demonstrate the role of secondary dispersal as a stabilizing mechanism in patterned dryland vegetation [71]. The advantage of such kernel-based approaches is that they provide a representation of the net effect of multiple runoff events, and allow simulations to be run at the coarse timescales corresponding to plant growth instead of the single-storm event needed in BOB-CELC. The disadvantage of such averaged representations of seed transport is that the variability between storms is ignored. To account for variations between storms, explicit simulations of runoff, seed dispersal, and ultimately germination and growth are required. These simulations are computationally intensive, but offer the prospect of process fidelity (at least with regards to time-scale matching between process and its representation in models). More mechanistic modeling, such as that performed in order to produce Figure 5, could also be coupled to explicit plant population models. By providing detailed hydrological information (e.g. soil moisture contents at the end of the storm as well as seed locations), mechanistic models of this nature not only act as a valuable basis for simulation, but offer considerable scope for testing predictions. Ultimately, models combining overland flow dynamics with seed dispersal and erosion could prove useful in the design of dryland restoration and revegetation strategies: an area where experimental trials are routinely implemented, but model assisted design remains uncommon [92–95].

## Conclusions

Recent developments in modeling seed dispersal and runoff generation in dryland ecosystems offer the potential for representing modes of secondary dispersal associated with overland flow. An extension to the existing CELC modeling framework was proposed (BOB-CELC) that showed qualitative agreement with dispersal behaviors reported in the literature. The framework provides a potential basis for exploring parsimonious representations of seed dispersal in patchy landscapes in which the final seed resting positions are largely tied to the vegetation distribution, as well as a fully mechanistic approach suitable for coupling to spatially and temporally explicit simulations. Despite these promising developments, there remains a clear need for targeted observations to reconstruct dispersal behavior in runoff in different patchy dryland ecosystems. Experiments targeting processes of transport initiation, trapping and termination, exploring the relative importance of and interactions between secondary wind and water dispersal, and linking dispersal processes to germination and growth success would be particularly informative. As particle tracking techniques [96, 97], high resolution imagery [98] and advances in LIDAR for mapping vegetation and water levels continue to improve [99–101], the time is ripe to coordinate experimental and theoretical developments.
